# Psychiatric disorders and clinical correlates of suicidal patients admitted to a psychiatric hospital in Tokyo

**DOI:** 10.1186/1471-244X-10-109

**Published:** 2010-12-13

**Authors:** Naoki Hayashi, Miyabi Igarashi, Atsushi Imai, Yuka Osawa, Kaori Utsumi, Yoichi Ishikawa, Taro Tokunaga, Kayo Ishimoto, Hirohiko Harima, Yoshitaka Tatebayashi, Naoki Kumagai, Makoto Nozu, Hidetoki Ishii, Yuji Okazaki

**Affiliations:** 1 Department of Psychiatry, Tokyo Metropolitan Matsuzawa Hospital, Tokyo, Japan; 2 Schizophrenia Research Team, Tokyo Institute of Psychiatry, Tokyo, Japan; 3 Faculty of Medicine, Tokyo Medical and Dental University, Tokyo, Japan; 4 Department of Psychogeriatrics, National Institute of Mental Health, National Center of Neurology and Psychiatry, Tokyo, Japan; 5 Mood Disorders Research Team, Tokyo Institute of Psychiatry, Tokyo, Japan; 6 Disabled Persons Programs Division, Bureau of Social Welfare and Public Health, Tokyo Metropolitan Government, Tokyo, Japan; 7 Tokyo Metropolitan Tama Comprehensive Center for Mental Health and Welfare, Tokyo, Japan; 8 Graduate School of Education and Human Development, Nagoya University, Nagoya, Japan

## Abstract

**Background:**

Patients admitted to a psychiatric hospital with suicidal behavior (SB) are considered to be especially at high risk of suicide. However, the number of studies that have addressed this patient population remains insufficient compared to that of studies on suicidal patients in emergency or medical settings. The purpose of this study is to seek features of a sample of newly admitted suicidal psychiatric patients in a metropolitan area of Japan.

**Method:**

155 suicidal patients consecutively admitted to a large psychiatric center during a 20-month period, admission styles of whom were mostly involuntary, were assessed using Structured Clinical Interviews for DSM-IV Axis I and II Disorders (SCID-I CV and SCID-II) and SB-related psychiatric measures. Associations of the psychiatric diagnoses and SB-related characteristics with gender and age were examined.

**Results:**

The common DSM-IV axis I diagnoses were affective disorders 62%, anxiety disorders 56% and substance-related disorders 38%. 56% of the subjects were diagnosed as having borderline PD, and 87% of them, at least one type of personality disorder (PD). SB methods used prior to admission were self-cutting 41%, overdosing 32%, self-strangulation 15%, jumping from a height 12% and attempting traffic death 10%, the first two of which were frequent among young females. The median (range) of the total number of SBs in the lifetime history was 7 (1-141). Severity of depressive symptomatology, suicidal intent and other symptoms, proportions of the subjects who reported SB-preceding life events and life problems, and childhood and adolescent abuse were comparable to those of the previous studies conducted in medical or emergency service settings. Gender and age-relevant life-problems and life events were identified.

**Conclusions:**

Features of the studied sample were the high prevalence of affective disorders, anxiety disorders and borderline PD, a variety of SB methods used prior to admission and frequent SB repetition in the lifetime history. Gender and age appeared to have an influence on SB method selection and SB-preceding processes. The findings have important implications for assessment and treatment of psychiatric suicidal patients.

## Background

Suicidal behavior (SB) is a major issue for mental health workers and often a cause of emergency treatment and psychiatric hospitalization. It also requires our special attention since it is usually seen as a salient sign of a high risk of suicide [1]. Psychiatric disorders have been ascertained to be a major causative factor for SB [1-3], and the treatment is expected to play an important role in reducing SB recurrence and preventing suicide [1].

A number of clinical investigations of suicidal patients have been conducted in medical or emergency service settings, which have increased our body of knowledge of the patient population, and improved our psychiatric practice for treating them. In contrast, the number of studies that have addressed suicidal patients admitted to a psychiatric hospital remains insufficient though these two patient populations are not identical, and may need to be treated differently. Only a portion of suicidal patients treated in medical or emergency settings were referred for psychiatric hospitalization [4-6]. It has also been asserted that suicidal patients admitted to psychiatric facilities exhibit characteristics that differ from those of patients who are primarily in need of medical treatment [4,7]. Therefore, investigation of the former group patients is needed to improve the treatment for them. In addition, this patient population should be an important target of studies since having both an SB experience and a history of psychiatric hospitalization are considered to be strong predictors of suicide [1,8,9].

To remedy the situation, we conducted extensive psychiatric evaluation of suicidal patients admitted to a psychiatric center in a metropolitan area of Japan by applying structured interviews. In the evaluation, we included the clinical characteristics that were dealt with as factors in theories of a pathway to suicide process [10,11], on the basis of which we previously showed a potential role of some pre-SB characteristics in the development of SB [12]. In the present study, we attempt to illuminate the clinical characteristics of this patient sample and their gender and age-relevance.

## Methods

### Subjects

This study was carried out at Tokyo Metropolitan Matsuzawa Hospital, a psychiatric center for psychiatric emergencies and other regional services in central Tokyo. The patients included in the study were those consecutively admitted with SB within a 20-month period from April 2006 to November 2007, and found to have exhibited SB during the week prior to their admission. The definition of "non-fatal suicidal behavior, with or without injuries" by de Leo, et al. [13] was applied in identifying the SB subjects. The selection criteria of the subjects were (1) age at admission equal to 20 years or more, (2) a hospital stay longer than 3 days, (3) absence of prominent mental retardation or organic brain damage, (4) fluent Japanese speaker, (5) exhibited an improvement that was judged to be sufficient to enable the subject to comprehend the study procedure and to undergo safely the study assessment during the hospital stay, and (6) provided the written informed consent for study participation or, in cases of involuntary hospitalization, additional consent was provided by the patient's family guardian.

### Assessment

The assessments conducted in this study were as follows.

#### (1) Suicidal Behaviors

Types of SBs immediately prior to admission and the frequency and period of SBs in the lifetime history of the subjects were recorded. The list of 16 SB types was made on the basis of that of suicide attempts used by Hosaka, et al. in the report of the 2004-2006 Japanese Ministry of Health, Labor and Welfare supported research. The types of SB such as self-cutting, overdosing or self-poisoning, self-strangulation, jumping from a height and attempting traffic death, were individually inquired in the first stage of assessment. The next stage was asking the period and the frequency of their occurrence in the lifetime history.

#### (2) Structured Clinical Interview for DSM-IV Axis I Disorders, Clinician Version (SCID-I, CV) [14] and Structured Clinical Interview for DSM-IV Axis II Personality Disorders (SCID-II) [15]

Psychiatric diagnoses of the subjects based on the Diagnostic and Statistical Manual of Mental Disorders, Fourth Edition (DSM-IV) [16], were determined by conducting SCID-I CV and SCID-II. These are clinician-administered semi-structural interviews for the evaluation of DSM-IV axis I and II disorders.

#### (3) Recent life events (RLEs) and life problems (LPs)

RLEs within 1 week, during 1 week to 1 month and during 1 month to 3 months prior to admission, and LPs before SB were recorded. 18 RLE types were selected from the item set of the studies of Paykel, et al. [17] and Heikkinen, et al. [18]. These were classified on empirical grounds into 3 domains: 9 RLEs in close personal relationships ((a) discord or conflict, (b) separation and (c) death, each of which was further classified in terms of whether the events referred to (1) spouse or partner, (2) other family members and (3) other close persons), 6 RLEs related to life situation ((c) troubles or changes in workplace or school, (d) loss of job or withdrawal from school, (e) financial problems, (f) moving house, (g) severe illness of any family member and (h) legal problems), and 3 RLEs related to health conditions ((i) physical illness, (j) mental illness and (k) pregnancy or abortion). In the analysis, the presence or absence of each domains of RLE during 3 months prior to admission was used. In the assessment of LPs, 4-point (absent, mild, moderate and severe) scales of the same items as those used for RLEs, were used. The LP items that were rated moderate or severe were used in the analysis.

#### (4) Suicide Intent Scales (SIS) [19]

SIS is a 20-item semi-structured instrument designed to record information concerning a suicidal person's wish to die at the time of a suicide attempt. In this study, a scale composed of the first 15 SIS items was used to rate the intensity of suicidal intent in terms of the circumstances and patient's reports of thoughts and feelings at the time of the attempt, and scales of Items 19 and 20 were used to rate the ingestion of alcohol and other drugs at the time of the suicide attempt, respectively.

#### (5) Beck Depression Inventory-II (BDI) [20] and Beck Hopelessness Scale (BHS) [21]

BDI is a widely used, 4-point, 21-item self-report scale developed for assessing depressive manifestations. BHS, a self-report scale for use in measuring hopelessness, is composed of 20 true-false items. In this study, these scales were used to assess the levels of depressive symptomatology and hopelessness of the subjects during 2 weeks prior to admission.

#### (6) Peritraumatic Dissociative Experiences Questionnaire (PDEQ) [22]

PDEQ involves an 8-item, 4-point scale devised for assessing dissociative symptoms during the action in question [22,23]. Originally, this scale was used for assessing the symptoms of Vietnam veterans during combat experiences. In this study, this questionnaire was used to measure the symptoms in SB as in the study of Cho, et al. [23].

#### (7) Overt Aggression Scale-Modified (OAS-M) [24]

OAS-M is 6- or 7-point, 9-item clinician-administered, semi-structured interview designed to measure various manifestations of 3 domains: aggression, irritability and suicidality of subjects. In this study, behavior within a week prior to admission was rated using this scale. In the analysis, scale scores of aggression, irritability and lethality of suicide attempt (item 7b) were used.

#### (8) History of abuse before the age of 18 years

To assess the history of abuse before the age of 18 year, a 3-point (absent, uncertain and certain), 7-item semi-structured interview was devised for use in this study. The items were intra- and extra-familial sexual abuse, intra- and extra-familial physical and verbal abuse and intra-familial neglect, which, except for sexual abuse, had lasted for longer than 1 month. Only items rated "certain" were used in the analysis.

The study assessment was performed principally over more than one interview since the inquiries were extensive, and might exhaust the subjects if conducted in a single session. Self-report scales were orally administered in the interviews. Information from medical records was also included in the study assessment.

The 10 interviewers were psychiatrists with more than 2 years of clinical experience. They had received 10 preparative educational sessions for the assessment and 3-5 on-site training sessions for SCID-I CV and SCID-II. All the study assessments were individually group-reviewed.

### Statistical analysis

Statistical tests were carried out to examine the effects of gender and age on the diagnoses and clinical characteristics, and included Chi-square tests, Fisher's exact tests, Mann-Whitney U tests and Spearman's rank order correlation coefficients. We applied a significance level of 0.05 and two-sided probability in exact tests and correlation analyses. Bonferroni correction was used in view of the number of statistical tests. SPSS version 16.0.2 statistical package (SPSS Inc., Chicago, IL, 2008) was used for the entire analysis.

This study was approved by the ethical committee of Tokyo metropolitan Matsuzawa Hospital on 28 Mar 2006.

## Results

Of a total of 3450 admissions to Tokyo Metropolitan Matsuzawa Hospital during the 20-month study period, 292 cases (280 patients) with SB were identified. 225 patients fulfilled the criteria (1)-(4). 157 (69.8%) of them (and their family guardian when necessary) gave consent to participate in the study, and 155 (68.9%) of them completed the assessment. 127 (81.9%) of the subjects were involuntarily admitted. The average (SD) duration of the period between admission and completion of the assessment was 25.7 (12.0) days.

There was no significant difference in ICD-10-based diagnoses in the hospital record or demographic and clinical characteristics presented in Table 1 between the subjects of this study and the 50 patients who were approached, but did not gave informed consent.

**Table 1:** Demographic and clinical characteristics of the subjects

	Male (N = 68)		Female (N = 87)		Total (N = 155)	
	N	%	N	%	N	%
Age at investigation (years)						
20-29	22	32.4	25	28.7	47	30.3
30-39	23	33.8	37	42.5	60	38.7
40-49	13	19.1	13	14.9	26	16.8
50+	10	14.7	12	13.8	22	14.2

Marital state						
Never married	48^a^	70.6	39	44.8	87	56.1
Cohabiting with spouse or partner	11	16.2	26	30.0	37	23.9

Living alone	34^b^	50.0	58	66.7	92	59.4

Education						
Less than high school	19	27.9	25	28.7	44	28.4
High school graduate	32	47.1	49	56.3	81	52.3
University (college) graduate	17	25.0	12	13.8	29	18.7

Unemployed	42	61.8	40	46.0	82	52.9

Referred after inpatient treatment for physical damage	14	20.6	8	9.2	22	14.2

Currently on psychiatric treatment	54	79.4	72	82.8	126	81.3

History of psychiatric hospitalization	38	55.9	52	59.8	90	58.1

Family history of mental disorder^c^	18	26.9	34	39.1	52	33.8

Family history of attempted or committed suicide^d^	10	14.7	16	18.4	26	16.9

^a ^The percentage of never married subjects for males was higher than for females (Chi-square = 10.29, df = 1, p = 0.001).

^b ^The percentage of living alone subjects for males was higher than for females (Chi-square = 4.40, df = 1, p = 0.036).

^c, d ^Among relatives within third degree consanguinity.

Table 1 shows the demographic and clinical characteristics of the subjects. The subjects consisted of 68 males and 87 females. Their average age (SD) was 36.5 (11.9) years old. 49 subjects (31.6%) started to exhibit SB at an age of 20 years or younger. The rates of unemployment and living alone were over 50%.

Table 2 shows the most frequent SBs that were exhibited by the subjects. The proportions of other SBs immediately prior to admission were lower than 3.3%. Over 60% of subjects had previously exhibited self-cutting and overdosing. The 25, 50 and 75 percentiles (range) of the total number of SBs in the lifetime history of the subjects were 3, 7 and 19 (1-141), respectively. The following associations of SBs with gender and age were found in the analyses where a significance level of 0.01 (0.05/5) was applied since statistical tests were conducted for each of the 5 SB methods shown in Table 2. The numbers of self-cutting and overdosing the subjects had experienced were greater for female subjects than for males (medians, ranges of females and males: 3, 0-132 and 1, 0-50 (p = 0.008, U = 2232.5, z = -2.67) and 2, 0-90 and 1, 0-100 (p = 0.003, U = 2142.5, z = -3.02), respectively). The number of self-cutting experiences had a significant negative rank-order correlation with age at investigation (-0.252, p = 0.002).

**Table 2:** Frequent suicidal behaviors (SBs) of the subjectsa

	SB prior to admission		SBs in the lifetime history			
			Method		**Mumber**^**b**^	
	N	%	N	%	Median	Range
Self-cutting	63	40.6	106	68.4	1	0-132
Wrist or forearm	41	26.5	96	61.9	1	0-100
Other part(s) of body	28	18.1	42	27.1	0	0-70

Overdosing	49	31.6	99	63.9	2	0-100
Prescribed psychotropics	43	27.7	95	61.3	1	0-100
Other prescribed medicine	4	2.6	5	3.2	0	0-30
OTC medicine	8	4.5	14	9.0	0	0-6

Self-strangulation	23	14.8	37	23.9	0	0-20
Hanging	12	7.7	25	16.1	0	0-20
Other self-strangulation	11	7.1	13	8.4	0	0-10

Jumping from a height	18	11.6	45	29.0	0	0-13

Attempting traffic death	16	10.3	27	17.4	0	0-20

SB: suicidal behavior

^a ^Significance level was set at 0.01 (0.05/5) since statistical tests were conducted for each of the 5 frequent SB methods shown in this table.

^b ^The SB immediately prior to admission was included.

6 DSM-IV axis I disorders and 10 axis II PDs of the subjects are exhibited in Tables 3 and 4. Affective disorders and anxiety disorders were presented by more than half of the subjects. It was found in the analysis that applied a significance level of 0.0083 (0.05/6) that subjects with anxiety disorders were younger than those without them (medians, ranges of the age: 32, 20-72 and 36, 21-76, respectively (p = 0.005, U = 2194.5, z = -2.78)). Most of the subjects had at least one PD. Borderline PD was the most frequent PD, and was exhibited by over 50% of the subjects. The analysis that applied a significance level of 0.005 (0.05/10) indicated that PDs, patients with which were younger than those without that PD were borderline PD and antisocial PD (medians, ranges of the age: 32, 20-55 and 39, 20-76 (p < 0.001, U = 1923.5, z = -3.76), and 31, 20-43 and 36, 20-76 (p = 0.002, U = 1606.5, z = -3.09), respectively).

**Table 3:** DSM-IV Axis I disorders of the subjectsa

	Male (N = 68)		Female (N = 87)		Total (N = 155)	
	N	%	N	%	N	%
Mood Disorders	36	52.9	60	69.0	96	61.9
Major Depressive Disorders	28	41.1	39	44.8	67	43.2
Dysthymic Disorder	0	0.0	5	5.7	5	3.2
Bipolar I Disorder	3	4.4	6	6.9	9	5.8
Bipolar II Disorder	4	5.9	8	9.2	12	7.7

Anxiety Disorders	28^b^	41.2	58	66.7	86	55.5
Panic Disorders	16	23.5	37	42.5	53	34.2
Specific Phobia	4	5.9	10	11.5	14	9.0
Social Phobia	3	4.4	6	6.9	9	5.8
Obsessive-Compulsive Disorder	7	10.3	6	6.9	13	8.4
Posttraumatic Stress Disorder	6	8.8	19	21.8	25	16.1
Generalized Anxiety Disorder	4	5.9	11	12.6	15	9.7

Substance-Related Disorders	24	35.3	35	40.2	59	38.1
Alcohol Use Disorders	15	22.1	29	29.9	41	26.5
Non-alcohol Use Disorders	12	17.6	16	18.4	28	18.1

Psychotic Disorders	22	32.4	19	21.8	41	26.5
Schizophrenia	18	26.5	13	14.9	31	20.0
Schizoaffective Disorder	3	4.4	0	0.0	3	1.9
Brief Psychotic Disorder	1	1.5	5	5.7	6	3.9

Eating Disorders	2	2.9	12	13.8	14	9.6
Anorexia Nervosa	0	0.0	2	2.3	2	1.3
Bulimia Nervosa	2	2.9	6	6.3	9	5.2
Eating Disorder NOS	0	0.0	4	4.6	4	2.6

Somatoform Disorders	0	0.0	7	8.0	7	4.5

Eating Disorder NOS: Eating Disorder not otherwise specified

^a ^Significance level was set at 0.0083 (0.05/6) since statistical tests were conducted for each of the 6 diagnostic groups shown in this table.

^b ^The percentage of subjects with anxiety disorders for males was lower than for females (p = 0.002, Exact test).

**Table 4:** DSM-IV personality disorders (PDs) of the subjectsa

	Male (N = 68)		Female (N = 87)		Total (N = 155)	
	N	%	N	%	N	%
Borderline PD	28^b^	41.2	58	66.7	86	55.5
Avoidant PD	21	30.9	28	32.2	49	31.6
Antisocial PD	22	32.4	20	23.0	42	27.1
Obsessive-compulsive PD	10	14.7	24	27.6	34	21.9
Paranoid PD	13	19.1	16	18.4	29	18.7
Schizoid PD	15	22.1	10	11.5	25	16.1
Narcissistic PD	7	10.3	11	12.6	18	11.6
Dependent PD	9	13.2	8	9.2	17	11.0
Schizotypal PD	5	7.4	7	8.0	12	7.7
Histrionic PD	3	4.4	8	9.2	11	7.1

Any PD	55	80.9	80	92.1	135	87.1

PD: personality disorder

^a ^Significance level was set at 0.005 (0.05/10) since statistical tests were conducted for each of the 10 PD types.

^b ^The percentage of subjects with borderline PD for males was lower than for females (p = 0.002, Exact test).

The proportions of the subjects who reported each of 3 domains of RLEs and LPs were RLEs and LPs in close relationships 69.7% and 60.0%, those in life-situation 61.9% and 63.2% and those in health conditions 18.1% and 52.9%, respectively. The proportions of those who reported discord or conflict, separation and death in close relationships were 62.6%, 22.6% and 9.0%, respectively. The following associations were found in the analysis that applied a significance level of 0.0167 (0.05/3). Female subjects reported RLEs and LPs in close personal relationships more frequently than males (Chi square = 10.91, df = 1, p = 0.001 and Chi square = 10.48, df = 1, p = 0.001, respectively). Those who reported life-situational RLEs or LPs were younger than those who did not (medians, ranges: 32, 20-69 and 36, 21-76 (p = 0.005, U = 2065, z = -2.83) and 32, 20-69 and 39, 21-76 (p = 0.001, U = 1866.5, z = -3.44), respectively).

The average (SD) of SIS suicidal intent scores was 11.7 (6.1). The proportion of subjects with high suicidal intent according to the criterion used by Skogman, et al. [6] (suicidal intent score > 18) was 13.5%. Alcohol and drug ingestion before SB occurred in 14.8% and 9.1% of the subjects, respectively. SIS alcohol and drug ingestion scores had a negative rank-order correlation with age at investigation (-0.316, p < 0.001 and -0.236, p = 0.003, respectively).

The averages (SDs) of BDI and BHS scores were 30.5 (12.3) and 13.1 (4.8), respectively. The proportions of depressive symptom severity levels based on BDI were minimal (0-9 points) 5.8%, mild (10-16 points) 8.4%, moderate (17-29 points) 29.7% and severe (30-63 points) 56.1%. Those of hopelessness severity levels based on BHS were mild (4-8 points) 14.8%, moderate (9-14 points) 35.5% and severe (15-20 points) 45.8%.

The averages (SDs) of the 3 OAS-M domain scores: aggression, irritability and medical lethality scores were 5.9 (7.0), 3.5 (2.8) and 1.8 (1.3), respectively. The average of the medical lethality score was almost "mild (2)". The analysis that applied a significance level of 0.0167 (0.05/3) indicated that the irritability score had a negative rank-order correlation with age at investigation (-0.246, p = 0.002). The average (SD) of the PDEQ score was 11.2 (7.1). The proportion of the subjects with any threshold dissociation symptom was 91.6% (142/155).

A history of any abuse before the age of 18 years was reported by 60.6% (94/155) of the subjects. The proportions of those who had experienced the 4 types of abuse were sexual abuse 16.8% (26/155), physical abuse 36.1% (56/155), verbal abuse 51.0% (79/155) and neglect 17.4% (27/155). It was found in the analysis that applied a significance level of 0.0125 (0.05/4) that sexual abuse was more common among female subjects than among males (24.1% (21/87) and 7.4% (5/68), respectively (p = 0.008, Exact test)).

## Discussion

Obviously, it is a characteristic of the studied sample that most of the patients had a psychiatric treatment history prior to index admission. The percentages of those who had currently been continuing outpatient treatment and those who had a history of psychiatric hospitalization were over 80% and over 50%, respectively while in the previous studies of suicidal patients in emergency settings, the proportions of those who had been receiving psychiatric treatment before admission were 50-69% [5,25,26]. The next noteworthy feature was a high proportion (over 80%) of the subjects who had a history of SB repetition. The figure was higher than those in previous studies of patients with suicide attempts or deliberate self-harm (DSH) [27] ranging from 25% to 65% [5,6,25,26,28,29]. In contrast, their physical conditions were not poor before admission as the lethality of their SB was typically mild, and only a small portion of the subjects (14%) received inpatient treatment for physical damage caused by SB.

The average age of the subjects of this study (37 years) was within the range of the previous studies in medical or emergency settings (26-42 years) [5,6,26,28-33]. The excess of female patients over males observed in this study was also common in previous studies [5,6,25,28-32]. High proportions of unemployment and living alone were also indicated as was in the review of Welch [33].

The SB methods recorded in this study were markedly different from those in the previous studies. Those in this study consisted of a variety of types, mainly not life-threatening ones such as self-cutting and overdosing while previous studies in medical settings reported that overdosing was the most common SB with ranges of 81-96% for DSH [29,31] and 29-93% for suicide attempts [5,25,26,32]. In particular, this study reported a higher rate of self-cutting than those in previous studies, which recorded rates of 4-12% for DSH [29,31] and 4-28% for suicide attempts [5,25,26,28].

The proportions of Axis I disorders found in the present study were not markedly different from the results from previous studies on suicide attempts [30] and DSH [29] that applied a structured diagnostic interview, and recorded affective disorders, substance-related disorders and anxiety disorders as major disorders. Exceptions were relatively high rates of psychotic disorders and anxiety disorders in this study. The excess of psychotic disorders could simply be explained by the fact that the field of this study was a psychiatric hospital. In contrast, the proportion of anxiety disorders higher than a little more than 20% of the previous studies that applied structured diagnostic interviews [29,30] might be specific of this study, and deserves further examination in new samples of psychiatric suicidal patients.

Concerning the PDs of SB patients, the importance of borderline and antisocial PDs has been emphasized [34] as this study sample showed high rates of both PDs. 2 previous studies reported a comparable rate of borderline PD among SB patients. Herpertz [35] reported that 52% (28/54) of inpatients that had exhibited more than 2 SBs had borderline PD. Söderberg [36] found that the proportion of borderline PD was 55% (35/64) among hospitalized suicidal patients by applying SCID-II. However, the studies of Haw, et al.[29,37], which used Personality Assessment Schedule as a self-report scale, showed only a low proportion (11%) of ICD-10 emotionally unstable PD, a subtype of which corresponds to DSM-IV borderline PD. On the other hand, the rate of antisocial PD in this study was comparable to that of Beautrais, et al. [30], and greater than those of Haw, et al. [29] and Söderberg [36]. These differences might be derived from the varied severity of psychiatric disorders among the samples in addition to the methodological diversity of PD assessment.

As in previous studies in medical settings [31,37,38], it was determined in this study that depressive symptoms are clinically important for suicidal psychiatric patients. The BDI and BSH scores were equal to or greater than those of previous studies [31,37]. The suicidal intent of the studied sample was within the range of those in previous studies [5,32,37].

The proportions of the studied subjects who reported RLEs and LPs were also comparable to those of previous studies on DSH patients [31,38] and on those who have attempted to commit or actually committed suicide [17,18] for the most part with the exception of a high percentage of perceived problems in mental health among subjects in this study. The previous studies [17,18,31,38] reported that the rate of SB- or suicide-preceding RLE or LP in close personal relationships was approx. 60%, and other major RLEs or LPs were those associated with occupation, financial conditions and physical health.

This study showed an association between troubles in the workplace or school before SB and younger age. Several studies [38-40] also reported that suicide or SB by young persons was frequently preceded by RLE in close personal relationships, lawsuits and troubles in the workplace or school. It is suggestive of life-cycle-relevance of SB-preceding RLEs and LPs that these troubles are common among young suicidal patients. However, the link reported by Haw, et al. [38] between an older age and experiencing physical difficulties was not observed in this study. In terms of gender difference in LPs, this study indicated that females more frequently experienced problems in close personal relationships as in the study of Haw, et al. [38].

Developmental factors, such as childhood and adolescent abuse, are assumed to have an influence on subsequent SB [41]. In this study, the proportion of suicidal patients that had experienced abuse at a young age was within the range of those in Japanese studies on various SB samples [12] while the figure was generally lower than those of the studies conducted in Western countries [41].

Lastly, limitations of this study need to be mentioned. First, this study is a retrospective and cross-sectional investigation, and is therefore hardly of use for determining causative factors or sequential processes of SB development. In particular, recall biases in evaluations concerning life-history factors such as abuse are inevitable. Second, PD diagnoses in this study, although based on a full application of SCID-II, could be improved. For instance, the PD diagnoses of this study were not exempted from the influence of coexisting axis I disorders that Zimmerman [42] pointed out. However, we consider that this influence is not so detrimental since the SCID-II was conducted after the subjects had recovered sufficiently to undergo extensive investigation.

## Conclusions

The present study has revealed high prevalence of affective disorders, anxiety disorders and borderline PD, and severe depressive symptomatology among psychiatric suicidal patients. A large variety of the SB methods used prior to admission and a high proportion of those who had a history of SB repetition appeared to be features of this studied sample distinct from those seen in medical and emergency service settings. This study also has confirmed gender and age-relevance of some SB-preceding life-problems and life events, which many previous studies on suicide victims and SB patients in emergency service settings identified. Further studies are needed to focus on those who appear with SB in psychiatric settings for the purpose of improving the services that they are subjected to.

## Competing interests

The authors declare that they have no competing interests.

## Authors' contributions

NH conceptualized and designed the study, collected the data, performed the statistical analysis, and drafted the manuscript. MI, AI, YO, KU, YI, TT and KI conceptualized and designed the study, collected the data. HH, YT, NK, MN and YO conceptualized and designed the study. HI performed statistical analysis. All authors read and approved the final manuscript.

## Pre-publication history

The pre-publication history for this paper can be accessed here:

http://www.biomedcentral.com/1471-244X/10/109/prepub
